# Levenshtein Distance, Sequence Comparison and Biological Database Search

**DOI:** 10.1109/tit.2020.2996543

**Published:** 2020-05-21

**Authors:** Bonnie Berger, Michael S. Waterman, Yun William Yu

**Affiliations:** Department of Mathematics and Electrical Engineering and Computer Science, Massachusetts Institute of Technology, Cambridge, MA 02139 USA, and also with the Department of Computer Science and AI Lab, Massachusetts Institute of Technology, Cambridge, MA 02139 USA; Quantitative and Computational Biology Section, Department of Biological Sciences, University of Southern California, Los Angeles, CA 90089 USA; Department of Mathematics, University of Toronto, Toronto, ON M5S 2E4, Canada, and also with the Department of Computer and Mathematical Sciences, University of Toronto at Scarborough, Toronto, ON M1C 1A4, Canada

**Keywords:** Levenshtein distance, sequence comparison, dynamic programming, similarity search, metric entropy

## Abstract

Levenshtein edit distance has played a central role—both past and present—in sequence alignment in particular and biological database similarity search in general. We start our review with a history of dynamic programming algorithms for computing Levenshtein distance and sequence alignments. Following, we describe how those algorithms led to heuristics employed in the most widely used software in bioinformatics, BLAST, a program to search DNA and protein databases for evolutionarily relevant similarities. More recently, the advent of modern genomic sequencing and the volume of data it generates has resulted in a return to the problem of local alignment. We conclude with how the mathematical formulation of Levenshtein distance as a metric made possible additional optimizations to similarity search in biological contexts. These modern optimizations are built around the low metric entropy and fractional dimensionality of biological databases, enabling orders of magnitude acceleration of biological similarity search.

## I. Introduction

In 1965 V. I. Levenshtein published “Binary codes capable of correcting deletions, insertions, and reversals,” a landmark paper [1] which in 1966 was translated into English [2] and is his most highly referenced work with over 10,000 citations. His work was based on an earlier paper of R. W. Hamming [3] that directly came from studying transmission of information and coding theory. Hamming’s methods handled error correction for one and two incorrect symbols in the transmission. Relevant to this discussion Hamming introduced what he called “a geometrical model” for the 2^*n*^ points in {0, 1}^*n*^ space. He introduced a distance or metric on this space by setting *D*(**x**, **y**) equal to the number of coordinates where *x*_*i*_ ≠ *y*_*i*_. This metric counts the number of substitutions needed to change **x** into **y** (or equivalently **y** into **x**) and is now called Hamming distance. Leven-shtein’s main interest is extending Hamming’s error correction results to include the case of allowing single letters to be inserted or deleted. His metric is the count of the minimum number of substitutions and single letter insertions or deletions to change **x** into **y**. This metric is known as Levenshtein distance, and it is clear that computing Levenshtein distance is more challenging than computing Hamming distance. Levenshtein also discusses counting reversals by which he means interchanging the order of two adjacent symbols in one sequence. In this paper, we will follow the developments of sequence distance metrics and sequence comparison in the biological sciences where it has proved to be crucially important. We will indicate the relevance of the methods of comparison to biology, including the complexity of computing the comparison statistics. Coding and information theory is not our focus here but it plays an important role in recent developments.

The advent of digital computers brought attention to a topic many of us still confront: how to correct spelling errors. Here is an early approach by Blair from 1960 that will illustrate the state of the art [4]. He notes that one could construct a file or vocabulary of all words and check if a typed word was in the file. If not in the file, it was misspelled although he points out that context may show a word which is in the file may not be correct as used in a certain context. His approach is based on similarity not distance, a distinction we will encounter later. Blair notes that a dictionary of all misspelled words is impractical. He instead groups vocabulary words by an algorithm to compute *r*-letter abbreviations to an *n*-letter word. Motivated by information theory, to compute his abbreviation he uses both letter position in the word as well as frequency of errors in that position and word length to compute similarity. This binning of words greatly shortens search of the vocabulary. Damerau approaches this problem by precomputing the vocabulary words as number of characters and a 28-bit character register [5]. A more modern approach in 1971 by Harrison already uses hashing [6].

Dynamic programming algorithms to compute Levenshtein distance and related statistics are versatile and widely used. They have independently appeared in many places. In the next section we will present some of those algorithms and while we will not attempt exhaustively to examine the origins of the methods, here are some references. In an influential 1983 book about this topic, Sankoff and Kruskal [7] present the following list of independent discoveries of dynamic programming algorithms for sequence comparison ranging from 1968 to 1975 ([8]–[16]). We add to this list Sellers’ 1974 paper [17]. Additionally, in [18] it was shown that edit distance and alignment similarity are dual; that is, given the weights for minimum distance alignment, there are weights for a corresponding similarity alignment and they have identical optimal alignments. This duality permits us to use distance and similarity interchangeably, as appropriate. After discussion of some of these alignment methods, we describe some recent advances in sequence database searches that run significantly more rapidly than the standard program BLAST [19], with little loss of accuracy. The new algorithm is based on metric entropy and fractal dimension [20].

## II. Biological Sequence Comparison

Many problems in computational biology can be recast as string problems. Here, we focus on biological sequence comparison and similarity search, but for a broader overview of computational genome analysis, we refer the reader to [21]. Additionally, for a more in depth dive into string analysis, Gusfield’s book is an excellent place to start [22].

### A. Dynamic Programming Algorithms for Sequence Comparison

Given two biological sequences (strings of DNA nucleotides or protein amino acids) of length *n*, the basic problem of biological sequence comparison can be recast as that of determining the Levenshtein distance between them. Biologists prefer to use a generalized Levenshtein distance where instead of simply counting the number of substitutions, insertions, and deletions, each operation will have a different cost depending on where in the string it is applied; i.e. common substitutions will have a lower cost than uncommon ones [23]. This generalization allows the Levenshtein distance to better serve as a proxy for the evolutionary distance between two sequences. Inserted or deleted letters (indels) are critical in biological sequence analysis and are also scored in the algorithms.

The full list of operations needed to transform one string into another then corresponds to a low-cost evolutionary path between the two. The dynamic programming (DP) algorithms we discuss here not only compute the Levenshtein distance but also output that list of operations. These are represented in the form of a pairwise ‘alignment,’ which inserts gap characters corresponding to insertion or deletion so that the transformed strings have Hamming distance equal to the Levenshtein distance between the original strings.


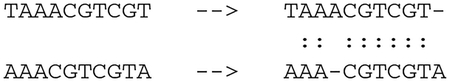


When only single-letter insertions and deletions are allowed the algorithms take time *O*(*n*^2^) to compute a matrix from which the optimal alignments can be extracted. It is not difficult to construct two sequences where the set of optimal alignments grows exponentially. In [24] this difficulty is handled by a declumping technique that recursively extracts those alignments along with all within a specified distance, where no pair of aligned letters appears on more than one alignment. For general indel length scoring, the algorithms take time *O*(*n*^3^). If the indel weights are an affine function of indel length, then there is an *O*(*n*^2^) algorithm that is widely used.

In biology, powerful inferences are made by comparing many sequences, say *m* sequences of length *n*, and the dynamic programming algorithms scale with time and storage *O*(*n*^*m*^), so the algorithms that are already time expensive for two sequences become impractical. In a study of regulatory sequences where the patterns are short and often different between sequences, [25] construct a table of all *k*-letter sequences and for each of the *n* sequences each *k*-word (also sometimes known in the field as a *k*-mer [26] or *k*-tuple [27]) receives a score of the best occurrence of that *k*-word in a specified neighborhood, for example with 2 mismatches of the *k*-word. This 1984 paper was the first use of word neighborhoods in biological sequence analysis.

BLAST is a highly cited program to search DNA and protein databases for evolutionarily relevant similarities of a target sequence to large databases of sequences. In fact for database search with BLAST, the program name has become a verb, as in “Just BLAST it.” The dynamic programming algorithm for this task is called local alignment and is often called the Smith-Waterman (S-W) algorithm [28]. This algorithm takes *O*(*n*^2^) time to accomplish this task and BLAST greatly reduces this search time with some possible loss in accuracy. BLAST uses the S-W dynamic programming algorithm as a subroutine in restricted regions of potentially significant matching. Before saying more about BLAST, we need to look more carefully at dynamic programming sequence alignment.

Levenshtein distance is a good proxy for the local mutations evolution uses to transform one string into another. However, much larger scale operations such as translocations (two distinct chromosomes mutate by exchanging parts), duplications, or deletions of long substrings also take place. To use a word processing analogy; biology occasionally cuts, copies, and pastes entire paragraphs. Luckily, large-scale changes happen rarely enough (relative to a three billion base pair human genome) that biologists choose to consider these phenomenon separately from the mutational changes caused by local edits. However, it is the presence of these large-scale changes that leads to the local alignment problem. Rather than the already expensive task of computing alignment between two strings, we now wish to find all the pairs of substrings, one taken from each string, that are both long and have low Levenshtein distance. Below, we show two different pairs of long local alignments of a single pair of strings.


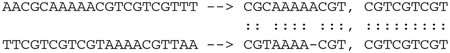


**Fig. 1.**
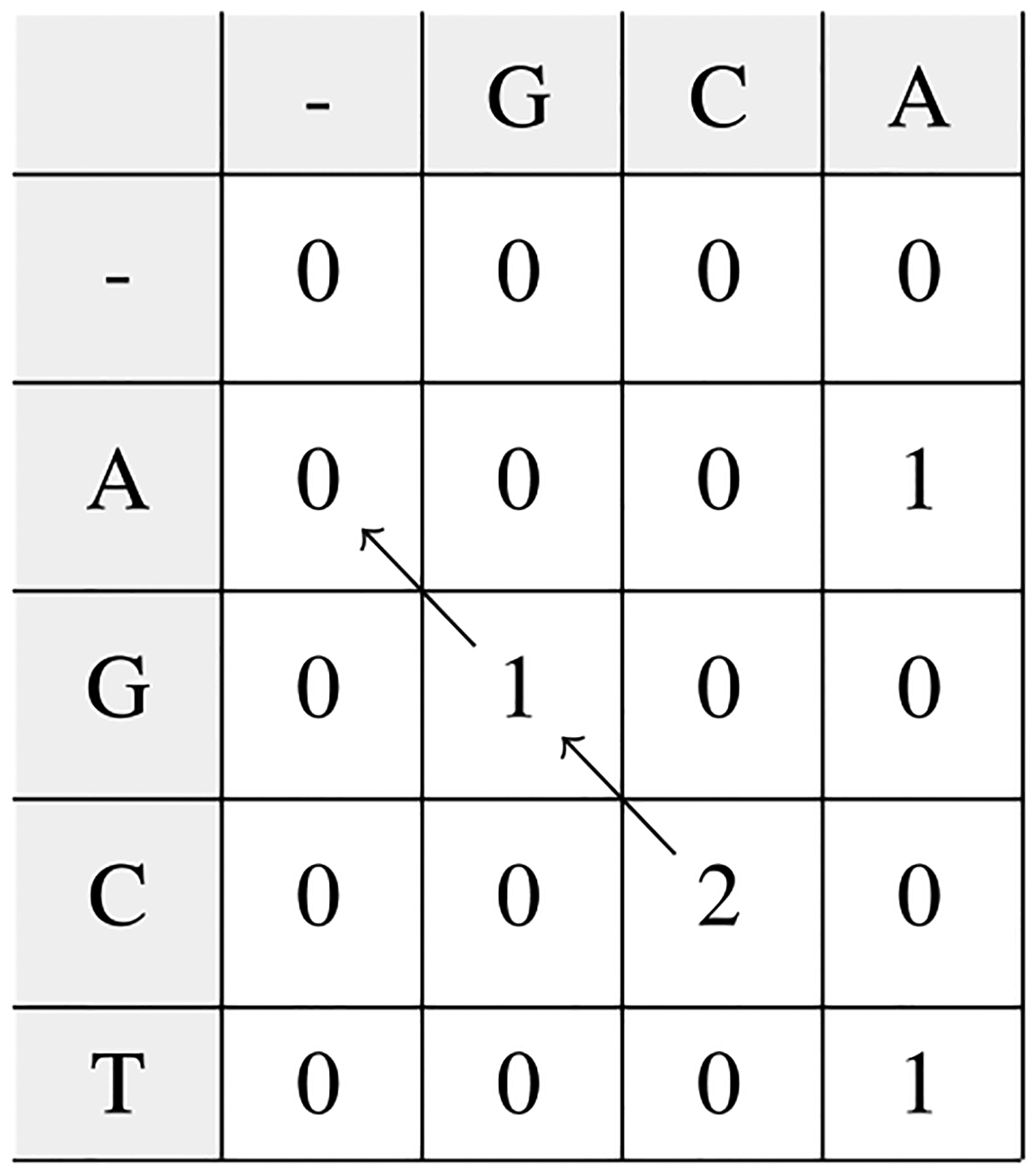
Smith-Waterman algorithm. Start with a scoring matrix. As an example, consider the alignment of two strings GCA and AGCT, where correctly aligned letters give a score of +1, substitutions a score of −1, and insertions or deletions a score of −2. The matrix is filled in recursively, with base case of 0’s in the leftmost column and top row. Moving to the right in the matrix corresponds to an insertion of a character from the left string, moving down corresponds to an insertion of a character from the top string, and moving diagonally down and to the right corresponds to either a correctly aligned letter or a substitution. A cell need only consider the three cells above and to the left of it. The score in a cell is the maximum score that can be achieved by coming from one of those three cells, with a floor of 0. After filling in the matrix, we need only scan the matrix for high scores, and then we can reconstruct the optimal path by tracing back from the maximum scoring cells. In this example, the optimal local alignment is GC to GC.

This problem is known as local alignment. When we state in the introduction that S-W solves local alignment optimally, we mean that the algorithm is guaranteed to return all such pairs of substrings in (asymptotically optimal) *O*(*n*^2^) time and space for strings of length *n*. The way S-W achieves this solution is depicted in Figure 1. Solving this problem with distance is obviously problematic as identical sequences have 0 distance whatever their length. Thus similarity is the key to the S-W solution as is the floor value of 0 which allows local alignments to begin and end anywhere.

### B. Fast Heuristics for Pairwise Sequence Comparison

While S-W is optimal for local alignment of two sequences, the quadratic runtime is too slow for many applications. Several heuristics to speed up pairwise local alignment were introduced in 1983 and 1985, and incorporated in the FASTA and FASTP software [29], [30], culminating in 1990 with the publication of BLAST (Basic Local Alignment Search Tool) [19].

Although the S-W scoring matrix efficiently codes for the highest scoring local alignments ending in every particular position, which is necessary to guarantee optimality, the bioinformatician generally cares about only the highest scoring alignments overall, rather than the highest-scoring alignments at every specific position. Thus, much of the information in the S-W scoring matrix is extraneous and can be safely discarded—or, more helpfully, never computed. The original program BLAST took advantage of this observation in a two step procedure. It is important to note that the underlying problem is to find significant matches of a query sequence in a large database of sequences. Critical to the process is the ability to compute the statistical significance of the S-W alignment score using the Gumbel extreme value distribution or Poisson approximation [31], [32], producing an ‘E-value’, which is interpreted roughly as the probability that a random string of that given length would produce a score that high. With this in hand, the algorithm takes each *k*-word of the query. Note that for protein sequences with a 20 letter alphabet, the word neighborhood surrounding each *k*-word is very large. Step one of the algorithm is to calculate all *k*-words which would constitute a statistically significant match, greatly reducing the computing time compared to the method of [25]. The next step is to search the large database for exact matches to this collection of statistically significant *k*-words. Finally extensions of found matchings are performed. Part of the advantage of this approach is that the neighborhood calculations need only be done for the query sequence.

For example, in the earlier example of a local alignment of two strings, let our query sequence be AACGCAAAAACGT CGTCGTTT, which has the following 6-mers: {AACGCA, ACGCAA, CGCAAA, GCAAAA, CAAAAA, AAAAAC, AAAACG, AAACGT, AACGTC, ACGTCG, CGTCGT, GTCGTC, TCGTCG, GTCGTT, TCGTTT}. Exact matches are very often statistically significant, though in principle, anything in the word neighborhood (e.g. CAAAAA –> CAAAAC) could be significant, depending on the generalized edit distance used. Then in order to find high-scoring local alignments, we need only find exact matches in the target seqeunce TTCGTCGTCGTAAAACGTTAA to significant matches in the word neighborhood of the query, and then perform S-W around those matches.

BLAST introduced many other computational optimizations to the pairwise sequence comparison problem. There are however many reviews already of BLAST and its variants [33]–[35], so we will not go into detail here. However, some of the other salient features include biology-specific augmentations. For example, there are many low-complexity repetitive regions in biological sequences; while strictly speaking, local alignments of low-complexity regions may be high scoring, they tend to pollute the results with many almost equivalent alignments. These low-complexity regions are important for a variety of neurodegenerative diseases [36], but have historically been problematic to characterize via alignment. Due to these many improvements, BLAST has been one of the prime workhorses of bioinformatics for decades, and was, as of a 2014 study, the 12th highest cited paper in any scientific field of all time [37].

Note, however, that with BLAST, we have begun to break the symmetry of the two sequences to be aligned. Although our goal is still to find optimal local alignments between two sequences, the algorithm performs the word-neighborhood search around only the query sequence. This can reduce the total amount of computation when we sequence the genome of a new species and wish to determine how it compares to another related species [38]; the word-neighborhood filtering is only done once. One important natural generalization of this problem is the multiple sequence alignment problem, where we have a collection of related genomes or proteins that we wish to simultaneously align to determine homology (similarity) [39], [40]. Here, though, we specifically consider the asymmetric alignment problem: biologists often have a specific (often short) query sequence that they wish to compare against a collection of many other existing sequences in a large database. BLAST is fast enough to serve in this function, but in so doing, it must perform a linear loop over each of the sequences that it scans in the database to find matching *k*-mers (although we used the more generic terminology ‘*k*-word’ earlier, ‘*k*-mer’ is the term bioinformaticians have settled on; *k* is typically 8–16 for nucleotide comparisons and 3–7 for amino acid comparisons.) However, the asymmetry between query and database can be further exploited to reduce runtime by preprocessing the database. One of the early examples of this was in 2002, when BLAT (BLAST-like alignment tool) [41] was introduced to speed up search by preindexing the database sequences for *k*-mers. Though BLAT is designed mostly as a BLAST-replacement, it presages the preprocessing-heavy techniques we will cover in the remaining sections of this paper.

### C. Shotgun Assembly vs. Alignment

The advent of ‘shotgun’ genomic sequencing technologies brought with it many text processing challenges. Shotgun sequencing is defined by not ‘reading’ the entire chromosome in one go; rather, it produces noisy data on ‘reads’, or random overlapping substrings of the chromosomal sequence [42]–[44]. The task of genome ‘assembly’ is to reconstruct the original genomic sequence from these random noisy substrings, generally by aligning and merging overlapping fragments [45]. It is closely related to the shortest common superstring problem [46], [47], though often the most likely sequence is not always the shortest superstring. Genome assemblers date back to the early 1980s [48] and have seen continual development and research in the last several decades [49]–[56]. It is not quite the sequence search or alignment problem, and so we do not focus on it in this review, but it is an important problem in its own right, making use of a whole host of algorithmic tools, including de Bruijn graphs [57], the Burrows-Wheeler Transform [58], the FM-index [59], and more [26].

However, given the successful assembly of the genome of a species—famously, some notable ones including the bacterium *Haemophilus influenzae*, yeast, fruit fly, and of course human genomes [60]–[64]—it is no longer necessary to perform a full assembly to analyze the genome of an individual member of the species. Instead, biologists use the already assembled genome as a template and align (or ‘map’) each of the reads to a similar location in that template. By keeping track of the variations from that template, it is possible to produce the genome of an individual at much lower cost [52], [65]. With personal and clinical genomics becoming ever more routine [66]–[69], mapping and and sequence comparison are very much in the limelight.

With Next-Generation Sequencing (NGS) technologies, read lengths became shorter (50–200 letters, or base pairs) yet more frequent [70]. This development makes read mapping a highly asymmetric variant of the standard sequence comparison problem. We now have billions of short reads that all need to be aligned to a single (relatively) static genome [71]. Additionally, BLAST and S-W are both capable of finding local alignments that are of much greater Levenshtein distance than we normally expect during NGS read-mapping; since read mapping is generally performed for sequences from the same species, not only are variations and mutations rarer, but we also readily accept not finding any reasonable alignment for a subset of the reads, labelling those reads ‘unmappable’ [72]. This combination of characteristics permits the development of a number of other read-mapping specific optimizations to the sequence comparison problem.

Classic methods for read mapping are indeed still versions of sequence similarity search like BLAST, but take advantage of a stable database and do significantly more clever preprocessing of it, in the form of suffix trees [73], [74], the FM-index [59], [75], and hash tables [76], [77]. The two most popular Illumina short-read mappers are BWA (Burrows-Wheeler Aligner) [78] and Bowtie 2 [79], though others such as SNAP [80] are also popular for specific settings. All of these methods gain considerable speed over their more general purpose sequence comparison brethren by indexing all the *k*-mers of the target genome in a fast, compressed data structure (through e.g. the FM-index or a hash table), so when a query read is input, they simply have to match *k*-mers in the query read to *k*-mers in the genome to find a starting point for a local alignment. Additionally, because read mapping admits lower sensitivity than sequence comparison search (it is not a travesty if a few reads do not get mapped), many mappers also employ a less sensitive ‘seed-and-extend’ strategy instead of a full local alignment: e.g. some early mappers (such as Bowtie 1 [81]) ignored insertions and deletions because those are expensive to include, although some mappers do provide the option of performing a full Smith-Waterman alignment (e.g. BWA-SW [78]).

We note here that there are several variants of the read mapping problem. The most common is ‘best-mapping’, which seeks to find the highest scoring local alignment of the entire read to the genome. Alternately, the ‘all-mapping’ task can be thought of as a threshold variant of the task, where given a threshold, we seek to find all local alignments of the read with score above that threshold. Finally, the ‘any-mapping’ task seeks only to find a single local alignment of the read above that threshold. We note that the all-mapping task is most similar in spirit to the sequence comparison task covered in the last section. Importantly, while we have presented the sequence comparison component of read mappers, read mapping is not a simple database search for approximately matching strings. Instead, modern read mappers will often make use of additional information about the genome sequencing machine’s confidence in its nucleotide measurements to build a probabilistic model of best matches. For more details on the vast field of read-mapping algorithms, we refer the reader to several other surveys [26], [82], [83].

## III. Return to Metric Roots

Much of our story in the previous section has had to do with specialization of methods to the particular bioinformatics variants of similarity search. Along the way, the mathematical generality of Levenshtein edit distance and Smith-Waterman local alignment has given way to fast heuristics based on matching *k*-mers. Especially once it came time for read mapping, the algorithms gained effectiveness partially through assumptions (both implicit and explicit) on the nature of particular sequencing technologies—indeed, as 3rd generation sequencing technologies (e.g. Pacbio [84], Oxford Nanopore [85], 10x [86]) become available, new mappers have been developed specifically for them [87]–[90].

However, Loh, Baym, and Berger [91] introduced another family of methods that leveraged the compressive structure of biological sequences (due to evolution) to reduce computation. In a later paper, also from Berger’s group, Yu et al. [20] observed and formalized that when data has low fractal dimension (defined below) [92], [93], one can often preprocess a database such that a sequence comparison query against it takes *O*(*S*) time, where *S* is the metric covering number of the database, a quantity closely related to both the metric (Kolmogorov) entropy [94]–[96] and Shannon entropy [97] of the database. We now discuss this result and how it can be used to accelerate general similarity search in many applications, from sequence comparisons to protein structure to mapping reads to astronomical spectra [91], [98]–[100].

### A. Metric Entropy and Fractal Dimension

A metric space (*X*,*d*) is any set of points *X* on which we define a distance function d:X×X→ℝ that satisfies the three metric properties; i.e. for any *x*, *y*, *z* ∈ *X*

1. *d*(*x*, *y*) = 0 ⇔ *x* = *y*
2. *d*(*x*, *y*) = *d*(*y*, *x*)
3. *d*(*x*, *z*) ≤ *d*(*x*, *y*) + *d*(*y*, *z*).

Consider a set *A* ⊂ *X*, where (*X*, *d*) is a metric space. Let *B*(*p*, *r*) = {*x* ∈ *X*|*d*(*x*, *p*) *< r*} be a metric ball of radius *r* around a point *p*.

*Definition 3.1*: The internal covering number $N_{\epsilon }^{int}(A)$ is the fewest number of points *a*_1_, …, *a*_*n*_ ∈ *A* such that the balls *B*(*a*_1_, *ϵ*), … *B*(*a*_*n*_, *ϵ*) cover A.

Note that it is also possible to define an external covering number where the ball centers are not necessarily in *A*, but can be drawn from any point in *X*. However, for our purposes, the internal covering number, where the ball centers *a*_*i*_ ∈ *A*, is easier to work with.

*Definition 3.2*: The metric (Kolmogorov) entropy $N_{\epsilon }^{ent}(A)$ is the largest number of points *a*_1_, …, *a*_*n*_ ∈ *A* one can find in *A* that are *ϵ*-separated, that is *d*(*a*_*i*_, *a*_*j*_) ≥ *ϵ* for all *i* ≠ *j*.

It is easy to see that $N_{2\epsilon }^{ent}(A)\leq N_{\epsilon }^{int}(A)\leq N_{\epsilon }^{ent}(A)$; that is to say, the metric entropy at *ϵ* and 2*ϵ* bound the internal covering number on both sides, so these concepts are roughly equivalent. As an aside, we use notation and definitions from Tao [96], [101] above. Note that some other sources define metric (Kolmogorov) entropy as $\text{log }N_{\epsilon }^{ent}(A)$ [95], to match more closely the standard definitions of Shannon entropy.

Kolmogorov entropy was first defined in the context of dynamical systems to quantify the amount of information needed to predict the future state of a trajectory to within *ϵ*-sided hypercubes [94], [95]. However, it can be intuitively understood as the amount of information needed to specify the location of a point $\bar{a}\in A$ to within tolerance *ϵ* when given knowledge of the set *A*. Given a minimum cardinality internal covering *B*(*a*_1_, *ϵ*), … *B*(*a*_*n*_, *ϵ*) of *A*, we can specify $\bar{a}$’s position to within error *ϵ* by producing *a*_*i*_ such that $\bar{a}\in B(a_{i},\epsilon )$. Thus, it takes $\text{log }N_{\epsilon }^{ent}(A)$ bits of information to specify $\bar{a}$’s position.

Thus one might ask, why define metric entropy without the logarithm? Consider the following: let *X* be the space of strings over a fixed alphabet with the Levenshtein distance as metric. Then given two strings *x*_1_ and *x*_2_, we can encode *x*_2_ in terms of *x*_1_ via the pairwise alignment, which we will denote via *x*_2_ − *x*_1_—i.e. we keep a list of the ordered insertions, deletions and substitutions needed to convert *x*_1_ to *x*_2_. For strings *x*_1_ = AAAACCCC and *x*_2_ = AAACCCTG, *x*_2_ −*x*_1_ = {(*del*, *, 4), (*sub*, *C*, 7), (*ins*, *G*, 8)}. Each edit can be encoded in *O*(log *n*), where *n* is the maximum of the lengths of *x*_1_ and *x*_2_, bits of information because the choice of indel or substitution takes constant additional information once the position in the string is specified. Let the Shannon entropy *H*(*x*_1_) of a string be the number of bits of information needed to specify that string (bounded above by 2*n* for a 4-letter alphabet). Given an encoding as an edit list, the Shannon entropy *H*({*x*_2_ − *x*_1_}) can be bounded by the number of edits times the logarithm of the string length. This implies that the Shannon entropy of the concatenation of two strings *H*({*x*_1_, *x*_2_}) ≤ *H*(*x*_1_) + *H*(*x*_2_ − *x*_1_).

**Fig. 2.**
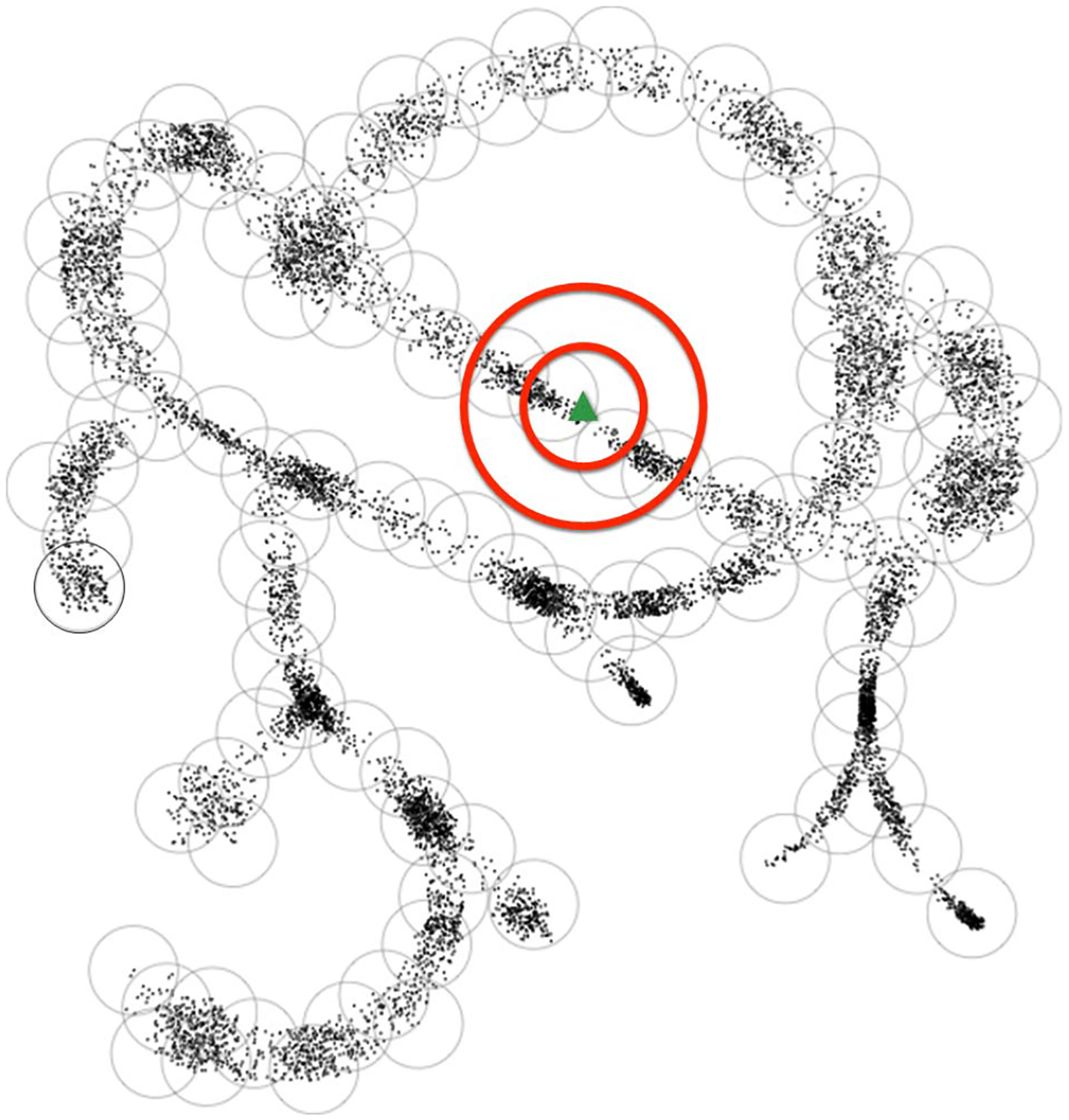
Cartoon illustration of coverings and low fractal dimension. In this example, points and balls are in a 2D space. The metric ball covers are the gray circles, the green triangle represents a search query *q*, the inner red circle is *B*(*q*, *s*), and the outer red circle corresponds to *B*(*q*, *s* + *r*). The red circles illustrate the desired search radius for similarity search and the needed wider search radius for finding any ball that might contain a point in the desired search radius. The number of metric ball coverings represent the covering number of the data, which is proportional to the Shannon entropy. The low fractal dimension is intuitively understood as there not being too many neighboring balls surrounding the one containing the query, and thus the covering looks tree-like. The theory generalizes to points in a high-dimensional space for which the balls would be hyperspheres. Figure taken from [20].

Hence, for |*A*| < ∞, *H*(*B*(*a*_*i*_, *ϵ*) ⊂ *A*) ≤ |*B*(*a*_*i*_, *ϵ*) ⊂ *A*|*ϵ* log(*n*) + *H*(*a*_*i*_). That is to say, we can encode the entire metric ball by encoding the center element, and then encoding the list of edits needed to form the other objects within the ball—note that depending on the distribution within the ball, the optimal encoding need not use the center as reference, but that still provides a useful upper bound. Given a suitable uniformity assumption on the number of elements within each of the balls of an internal cover on *A* (i.e. all balls have size *θ*(*K*), for some constant *K*) and the lengths of the *a*_*i*_’s (all bounded by *n*), this analysis shows that the covering number is proportional to the Shannon entropy of the dataset. As such, defining metric entropy without the logarithm makes it (roughly) proportional to the Shannon entropy under these circumstances. We will soon see that when these conditions are satisfied, a straightforward cluster-based similarity search has runtime roughly linear in the metric entropy, or covering number (Figure 2.).

For the coming analysis, we will also need an additional tool from fractal geometry [92].

*Definition 3.3*: The fractal (Minkowski) dimension

$$d(A)=lim_{\epsilon \to 0}\frac{\text{log}\left(N_{\epsilon }^{int}(A)\right)}{\text{log}(1/\epsilon )}.$$

However, for a finite set of points, the Minkowski dimension is always 0. Therefore, we also define the following.

*Definition 3.4*: The local fractal dimension at a scale *r* and range *s* is

$$d(A,r,s)=\underset{a\in A}{\text{max}}\text{ log}\frac{\left|B(a,r+s)\right|/\left|B(a,r)\right|}{(r+s)/r}.$$

Intuitively, we use fractal dimension to describe the intrinsic scaling behavior of our set *A* with respect to the number of points contained in a metric ball as we vary the radius of the ball. When the fractal dimension is low, that implies that all of the set *A* lives close to a low-dimensional manifold, and is not dense in the space *X*, allowing us to avoid the curse of dimensionality which often troubles similarity search [102].

### B. Clustered All-Neighbors Similarity Search

Building upon the aforementioned concepts, Yu et al. [20] introduced to bioinformatics a clustered all-neighbors similarity search, which they called ‘entropy-scaling search.’ Entropy-scaling search for queries in biological databases is often orders of magnitude faster than existing methods.

Consider now a simple organization of the database of items *A* into clusters corresponding to a minimal internal covering of metric balls of fixed radius *r* around points *a*_1_, …, *a*_*S*_; i.e. a simple metric search tree [103], [104]. Given a query point *q* and a query radius *s*, we wish to find *B*(*q*, *s*) ⊂ *A*. By the triangle inequality, we know that

$$B(q,s)\subset \underset{i:d\left(a_{i},q\right)\leq r+s}{\cup }B\left(a_{i},r\right).$$

We can thus find *B*(*q*, *s*) by first searching for ball centers, and then searching only within nearby balls. Then, given some uniformity assumptions on the sizes of the clusters (i.e. that the largest cluster has no more than a constant multiple of the number of elements of the smallest cluster), the runtime (in pairwise comparisons) of a clustered all-neighbors similarity search is:

$$O\left(\underset{\text{metric entropy}}{\underset{︸}{S}}+\overset{\text{output size}}{\overset{︷}{\left|B(q,s)\right|}}\underset{\text{scaling factor}}{\underset{︸}{{\left(\frac{s+2r}{s}\right)}^{d(A,s,s+2r)}}}\right).$$

When the local fractal dimension is small, the runtime is dominated by the metric entropy of the database, which is much less expensive than a naive analysis of metric search trees would suggest [20]. We visualize this property in a cartoon of low fractal dimension in 2D at the scale of the search (Figure 2).

This result immediately lends itself to application for not only finding all strings in a database within some Levenshtein distance of a query string, but indeed similarity search in any metric space with low fractal dimension, which biological data exhibits [20]. However, this analysis is predicated upon having a metric distance. Unfortunately, the E-values that are the primary output of BLAST are not metrics. Still, because they are derived from S-W alignment scores, themselves often based on Levenshtein distance, we can work around the lack of an exact Triangle Inequality by simply setting the initial ball center search to a small constant-factor wider radius around the query sequence. In so doing, at the cost of some expensive preprocessing, it is possible to achieve *orders of magnitude* acceleration of BLAST variants—including genomic, protein, and metagenomic sequence comparison—when the database is much larger than the query sequence [20], [91], [98] (See http://cb.csail.mit.edu/cb/gems/ for some open source software examples). Notably, as this approach is built around preprocessing the database, it is designed to accelerate existing similarity search tools, taking advantage of the optimizations existing methods have built for the sequence comparison primitive. For the problem of read all-mapping, which is based directly around Levenshtein distance, the same approach is simpler to analyze and achieves in practice 10x to 4,700x runtime improvement of existing read mappers [99]. Similar results built upon on this work can be shown for hierarchical metric search trees [100] and in other non-sequence applications, such as stellar spectra [100] and small molecule subgraph alignment (demonstrating a 150x speedup of the last) [20].

## IV. Conclusion

As bioinformatics data continues to grow in volume and ease of acquisition, it is essential to develop more sophisticated algorithms for data processing [105]. Levenshtein published his landmark paper over half a century ago [1], forming the foundation of sequence comparison search. Although some modern bioinformatics heuristics using *k*-mer matching have partially supplanted the direct optimization of Levenshtein distance through dynamic programming, his formulation of metric string distance remains relevant to this day and a source of inspiration for active research.
